# Barriers to using skilled birth attendants’ services in mid- and far-western Nepal: a cross-sectional study

**DOI:** 10.1186/1472-698X-13-49

**Published:** 2013-12-23

**Authors:** Bishnu Choulagai, Sharad Onta, Narayan Subedi, Suresh Mehata, Gajananda P Bhandari, Amod Poudyal, Binjwala Shrestha, Matthews Mathai, Max Petzold, Alexandra Krettek

**Affiliations:** 1 Department of Internal Medicine and Clinical Nutrition, Institute of Medicine, Sahlgrenska Academy at University of Gothenburg, Gothenburg, Sweden; 2 Department of Community Medicine and Public Health, Institute of Medicine at Tribhuvan University, Kathmandu, Nepal; 3 Nepal Public Health Foundation, Kathmandu, Nepal; 4 World Health Organization, Geneva, Switzerland; 5 Centre for Applied Biostatistics, Sahlgrenska Academy at University of Gothenburg, Gothenburg, Sweden; 6 Nordic School of Public Health NHV, Gothenburg, Sweden

**Keywords:** Antenatal care, Delivery care, Utilization, Skilled birth attendant, Barrier, Nepal

## Abstract

**Background:**

Skilled birth attendants (SBAs) provide important interventions that improve maternal and neonatal health and reduce maternal and neonatal mortality. However, utilization and coverage of services by SBAs remain poor, especially in rural and remote areas of Nepal. This study examined the characteristics associated with utilization of SBA services in mid- and far-western Nepal.

**Methods:**

This cross-sectional study examined three rural and remote districts of mid- and far-western Nepal (i.e., Kanchanpur, Dailekh and Bajhang), representing three ecological zones (southern plains [Tarai], hill and mountain, respectively) with low utilization of services by SBAs. Enumerators assisted a total of 2,481 women. All respondents had delivered a baby within the past 12 months. We used bivariate and multivariate analyses to assess the association between antenatal and delivery care visits and the women’s background characteristics.

**Results:**

Fifty-seven percent of study participants had completed at least four antenatal care visits and 48% delivered their babies with the assistance of SBAs. Knowing the danger signs of pregnancy and delivery (e.g., premature labor, prolonged labor, breech delivery, postpartum hemorrhage, severe headache) associated positively with four or more antenatal care visits (OR = 1.71; 95% CI: 1.41-2.07). Living less than 30 min from a health facility associated positively with increased use of both antenatal care (OR = 1.44; 95% CI: 1.18-1.77) and delivery services (OR = 1.25; CI: 1.03-1.52). Four or more antenatal care visits was a determining factor for the utilization of SBAs.

**Conclusions:**

Less than half of the women in our study delivered babies with the aid of SBAs, indicating a need to increase utilization of such services in rural and remote areas of Nepal. Distance from health facilities and inadequate transportation pose major barriers to the utilization of SBAs. Providing women with transportation funds before they go to a facility for delivery and managing transportation options will increase service utilization. Moreover, SBA utilization associates positively with women’s knowledge of pregnancy danger signs, wealth quintile, and completed antenatal care visits. Nepal’s health system must develop strategies that generate demand for SBAs and also reduce financial, geographic and cultural barriers to such services.

## Background

The services of skilled birth attendants (SBAs) include antenatal care (ANC) as well as delivery and postnatal care. Such services are critically important for reducing maternal and neonatal mortality because they provide timely delivery of obstetric and newborn care when life-threatening complications arise [1]. Globally, the maternal mortality ratio (MMR) decreased from 400 maternal deaths per 100,000 live births in 1990 to 210 in 2010 [2]. Low-income countries account for 99% (284,000) of all maternal deaths worldwide and a majority of deaths occur in sub-Saharan Africa (162,000) and Southern Asia (83,000) [2].

The total fertility rate in Nepal declined, contraceptive use increased, and MMR decreased between 1990 and 2010 [3]. Currently, Nepal has 2,875 nurse-midwives, 16,506 other health professionals with some midwifery competencies, and 3,000 community health workers with some midwifery training [3]. Nevertheless, disparities persist regarding access to and utilization of maternal health care between ethnic groups, among people with different education and income levels, and between geographic areas [4].

Improving women’s SBA utilization rate during childbirth is an important component of the Millennium Development Goals (MDGs) [5]. The World Health Organization (WHO) established international targets for SBA-assisted births (i.e., 80% by 2005, 85% by 2010, and 90% by 2015) [6], but countries with high MMR, such as Nepal, should achieve at least 40% SBA-assisted births by 2005, 50% by 2010, and 60% by 2015 [6]. SBA utilization during delivery has increased steadily in Nepal, from 9% in 1996 to 36% in 2011 [4,7]. Despite progress in achieving MDG targets regarding maternal and child health, such progress has been unequal in Nepal’s administrative regions. In the mid- and far-western regions, SBA utilization during deliveries is 28.7% and 30.6%, respectively, compared to higher utilization in Eastern, Central, and Western Nepal (42.0%, 35.9%, and 37.8%, respectively) [4]. Maternal mortality in Nepal is currently 281 deaths per 100,000 live births) [8], possibly due to a high prevalence of home births and low utilization of SBAs [9]. The main causes of maternal mortality in Nepal are postpartum hemorrhage (32%), hypertensive disorder of pregnancy (25%), and abortion (13%) [10].

Notably, Nepal’s mid- and far-western regions have been slower in reducing infant mortality. The national infant mortality rate (IMR) is 48 deaths per 1,000 live births, compared to 97 and 74 in the mid- and far-western regions, respectively [8]. Moreover, neonatal mortality decreased more slowly than IMR during the past 15 years. Indeed, the proportion of neonatal deaths increased from 63% of all infant deaths in 1996 to 72% in 2011 [4]. One MDG 5 indicator (i.e., improvement in skilled attendance at birth) has increased at a slower pace; consequently, Nepal is unlikely to achieve MDG 5 [11]. Because utilization of SBA services contributes importantly to reducing maternal and neonatal mortality, Nepal needs to identify barriers and develop strategies for improving access and utilization of such services in the mid- and far-western regions.

In 2005, the Government of Nepal introduced the Maternity Incentives Scheme to increase the use of professional care at childbirth [12] and provide each woman who delivers in a health institution a fixed amount of money to cover transportation costs, based on ecological region. Thus, women in the mountainous, hilly, and plain Tarai regions receive 1,500, 1,000, and 500 Nepalese rupees (NRPs) (1 US$ ≈ NRP 96 in August 2013), respectively, for each birth that takes place in a health institution [13].

ANC offers an important opportunity for healthcare providers to inform women about the advantages of delivering their babies with the help of an SBA. Moreover, ANC teaches pregnant women about the danger signs of pregnancy, enabling them to recognize early symptoms and go to a health facility as soon as possible. For women with normal pregnancies, WHO recommends a minimum of four ANC visits, ideally at 16, 24–28, 32, and 36 weeks [14]. In low-income countries, about 68% of mothers complete at least one ANC visit and almost 60% complete four or more visits [15]. Increased availability of SBA challenges healthcare providers in mid- and far-western Nepal, where SBA-attended births total less than 20% [16]. Our study investigated barriers to ANC and delivery care, two important components of SBA services.

## Methods

### Study setting

**Figure 1:**
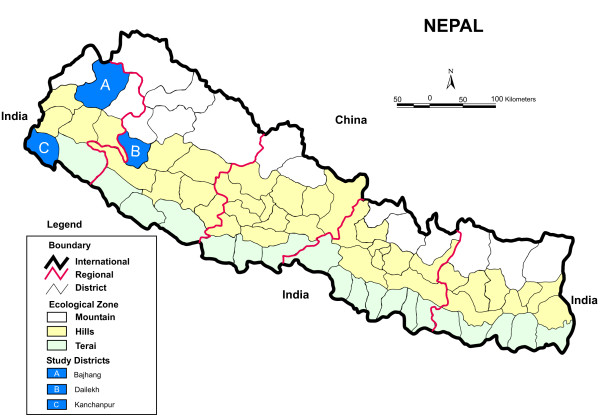
**Map of Nepal showing study districts.** The map shows three districts (i.e., Bajhang, Dailekha, and Kanchanpur) in mid- and far-western regions that have low SBA utilization. Modified from Wikipedia (http://en.wikipedia.org/wiki/File:Nepal_districts.png).

Our study focused on the mid- and far-western regions of Nepal because their utilization of SBA services is lower compared to other regions [17]. The human empowerment index shows marked disparities across Nepal’s five development regions. The index is constructed by merging available social, economic, and political indicators into a composite index of empowerment [18]. The central development region (0.497) scored highest, followed by the eastern development region (0.486) [18]. In contrast, the mid- and far-western regions scored lowest, about 15% below the national average [18]. Developmentally, the lowest-scoring regions lag in all dimensions of human empowerment. We conducted this study in three purposively selected districts (Figure 1) to represent all three ecological zones: mountain (Bajhang district), hill (Dailekh district), and plain Tarai (Kanchanpur district).

Administratively, we divided the study districts into “Ilaka” comprising three to five village development committees (VDCs) and municipalities. VDCs are the basic politico-administrative units of Nepal. Bajhang and Dailekh each contain 48 VDCs, 55 VDCs and 1 municipality, whereas Kanchanpur contains 19 VDCs and 1 municipality.

### Study design and participants

**Figure 2:**
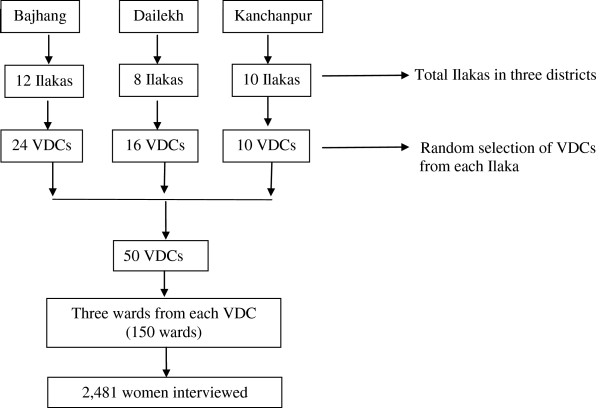
**Sampling procedure.** Administratively, the study districts are divided into “Ilaka” comprising three to five village development committees (VDCs) and municipalities. VDCs are the basic politico-administrative units of Nepal. Each VDC is divided into nine wards.

Our cross-sectional study used two-stage cluster sampling to obtain a representative sample of women who had delivered a baby within the preceding 12 months. Bajhang, Deilekh, and Kanchanpur contain 12, 8, and 10 Ilakas, respectively. We randomly selected two VDCs from each Ilaka in Bajhang and Dailekh and, due to the larger population and higher number of deliveries per VDC, one VDC from each Ilaka in Kanchanpur. Thus, we sampled 50 VDCs—24 from Bajhang, 16 from Dalikeh, and 10 from Kanchanpur (Figure 2). Next, we used systematic random sampling to select three wards from each sample VDC. From the selected wards, we interviewed all women with a child younger than one year of age. With an estimated coverage of SBA, P = 15%, a design effect of 2.0, allowable error 12% of P, and a 95% confidence interval, we calculated a sample size of 3,030 women. However, only 2,481 women were available for interview in the sampled clusters.

### Data collection

We used a structured questionnaire to collect data from women who had delivered a baby within the 12 months preceding data collection. We pretested the questionnaire in two Kathmandu VDCs (i.e., Pharping and Setidevi) located in peripheral areas of the Kathmandu district and having a rural scenario similar to the study sites. The questionnaire covered socioeconomic information including education, occupation, ethnicity, religion, and economic status; SBA availability; and women’s knowledge of the danger signs and health problems that occur during pregnancy and delivery. To assess women’s knowledge about the danger signs of pregnancy and delivery, we asked if they knew the danger signs and then asked the women to identify those signs. We recorded all responses.

Data collection was conducted between 6 May and 2 June 2011. Altogether, 48 enumerators with public health background (i.e., bachelor of public health or equivalent) were mobilized in coordination with their respective district’s health offices. All enumerators completed a five-day training session that included instructions for conducting data collection and an explanation of each section of the collection tool that was developed specifically for this study. Enumerator training also included a pre-test of the collection tool. All enumerators and supervisors received a field manual.

Enumerators met the health institution in charge as well as other health workers in the respective VDCs. To prepare a list of mothers with a child younger than one year of age, the enumerators used the Bacille Calmette-Guerrin (BCG) immunization register and the reporting form used by maternal and child health (MCH) workers and village health workers [19]. BCG immunization coverage is 100% and 99% in mid- and far-western regions of Nepal, respectively [20]. MCH and village health workers use a single form to report all services they provide in the community, including the MCH services. Thus, combining the BCG register with the reporting form used by MCH and village health workers yielded a complete list of respondents for our study. Female community health volunteers gathered mothers at a convenient location for individual face-to-face interviews. We mobilized six teams of enumerators, each containing eight members. To conduct the questionnaire/interview individually, we also ensured the availability of an enumerator for each woman. To ensure that no eligible woman was omitted, the enumerators visited all households included in the records of the combined BCG register and MCH reporting form.

### Study variables

The two dependent variables in this study were utilization of ANC and delivery services. The independent variables included socioeconomic status; accessibility of SBAs, as measured by distance to health facility; and knowledge of the danger signs of pregnancy and delivery (i.e., premature labor, prolonged labor, breech delivery, cord prolapse, postpartum hemorrhage, maternal injuries, severe headache, convulsion, high fever, foul-smelling discharge, and no movement of baby in the womb). We used principal component analysis to determine household economic status by calculating a wealth index based on household assets. Education was defined as illiterate (unable to read and write), informal (learning not connected to formal schools), primary school (grades 1–5), secondary school (grades 6–10), and intermediate and above (higher secondary, grades 11 and above).

WHO defines an SBA as “an accredited health professional—such as a midwife, doctor or nurse—who has been educated and trained to proficiency in the skills needed to manage normal (uncomplicated) pregnancies, childbirth and the immediate postnatal period, and in the identification, management and referral of complications in women and newborns” [21]. In our study, SBAs were health workers who had completed 15 or more months of training in nursing or general medicine and who were registered by their respective professional councils. Maternal and child health workers had completed six months of training in maternal and child health.

### Statistical analyses

We entered data obtained from the structured questionnaire into EpiData 3.1 and then transferred the information to SPSS Version 17.0 for analysis. Next, we performed simple and multiple logistic regressions to determine the association between delivery, ANC service utilization, and background variables (e.g., education, occupation, wealth quintile, distance to health facility, and knowledge of danger signs). We calculated the variance inflation factor (VIF) of the variables to check collinearity prior to inclusion in the regression. We identified no problem of collinearity among the independent variables (highest VIF, 1.256) that would bar them from the analysis. Our multiple logistic regression analyses included all independent variables that were significant at the 10% level in the simple regression analyses. Statistical significance was set at p < 0.05.

### Ethical considerations

After explaining the nature of the study, its rationale, and the extent of participant involvement, enumerators sought written informed consent from every participant. Informed consent and interviews were conducted with due respect to providing privacy and helping respondents feel secure in expressing their answers. A witness read the informed consent form to illiterate women and those who consented to participate applied their thumbprint on the questionnaire. We also received the signature of the witness in the consent form. The Nepal Health Research Council and the WHO (Geneva, Switzerland) granted ethical approval for this study.

## Results

### Sociodemographic characteristics

Most respondents were Hindu (98%). The main ethnic groups were Brahmin/Chhetri (61%), Dalit (untouchable caste, 20%), and Janajati (indigenous group, 13%) (Table 1). Three-quarters (75%) of the respondents were 20–35 years of age (median age = 24 years). Only 8% of respondents had attended higher secondary school or above, but more than three-quarters (76%) were literate. Agriculture was the major occupation for three-quarters (75%) of the study population (Table 1). The median age at marriage was 17 years, and more than two-thirds (70%) of the mothers gave birth to their first child before reaching 20 years of age (data not shown). At the time of the study, two of every five respondents (41%) had three or more children (Table 2).

**Table 1:** Sociodemographic characteristics of the study population

**Variables**	**Number of women**	**Percentage**
**Age groups (years)**		
< 20	553	22.3
20-35	1850	74.6
>35	78	3.1
**Ethnicity**		
Brahmin/Chhetri	1515	61.1
Dalit	490	19.7
Janajati	323	13.0
Religious minorities	153	6.2
**Education**		
Illiterate	591	23.8
Informal	915	36.9
Primary	315	12.7
Secondary	464	18.7
Intermediate and above	196	7.9
**Occupation**		
Agriculture	1901	75.3
Service	57	3.6
Business	42	1.7
Wage laborer	36	1.5
Housewife	398	16.0
Other	47	1.9
**Wealth quintile**		
Lowest	503	20.3
Second	555	22.4
Middle	432	17.4
Fourth	499	20.1
Highest	492	19.8

Results are shown for 2,481 women from the mid- and far-western regions of Nepal.

**Table 2:** Maternal health characteristics of the study population

**Variables**	**Number of women**	**Percentage**
**Knowledge of danger signs**		
No	1,204	48.5
Yes	1,277	51.5
**Frequency of ANC visit**		
< 4 times	1,059	42.7
≥ 4 times	1.422	57.3
**Utilization of delivery services**		
No	1,285	51.8
Yes	1,196	48.2
**Distance to health facility**		
>30 min	1,691	68.2
≤30 min	790	31.8
**Parity**		
One	784	31.6
Two	686	27.7
Three or more	1,011	40.7

### Utilization of maternal health services

Among 2,481 respondents, 88.3% completed at least one ANC visit and 57.3% completed four or more visits (Table 2). Assistant nurse-midwives were the primary providers of ANC services (67%), followed by maternal and child health workers (18%) (data not shown). SBAs assisted 48% of the most recent deliveries. One percent of women delivered their babies while en route to a health facility.

Respondents reported that the major reasons for seeking SBA services for their most recent deliveries included safe delivery (70%) and better management of potential complications (26%). The main reasons that women did not seek SBA services included distance to a health facility (45%) and inadequate transportation (21%). Nearly one- third of our respondents (32%) had to travel more than 30 min to reach the nearest health facility (Table 2). Only 51% of respondents knew at least one danger sign of pregnancy and delivery (Table 2).

Education level, wealth quintile, knowledge of the danger signs of pregnancy and delivery, and distance to a health facility associated positively with four or more ANC visits. Mothers’ age did not associate significantly with ANC and delivery service utilization. We observed a significant association between knowledge of delivery danger signs and ≥4 ANC visits (OR = 1.71, 95% CI: 1.41-2.07). Respondents living within 30 min of a health facility were 1.4 times more likely to use ANC services (OR = 1.44, 95% CI: 1.18-1.77) compared to those who had to walk more than 30 min (Table 3).

**Table 3:** Determinants of antenatal care service utilization

**Characteristics**	**Antenatal care utilization (< 4 times and ≥ 4 times)**	
	**Bivariate** **OR (95% CI)**	**Multivariate** **OR (95% CI)**
**Education**		
Illiterate	1	1
Informal education	1.54 (1.22–1.95)*	1.39 (1.10-1.77)*
Primary school	1.81 (1.34–2.45)*	1.38 (1.01-1.90)*
Secondary school	2.85 (2.12–3.78)*	1.94 (1.43-2.63)*
Intermediate and above	3.49 (2.35–5.17)*	2.41 (1.55-3.75)*
**Occupation**		
Agriculture	1	1
Service	1.63 (0.99-2.68)	1.05 (0.61-1.80)
Business	1.95 (0.92-4.15)	1.34 (0.61-2.93)
Wage laborer	1.33 (0.63-2.83)	1.36 (0.63-2.94)
Housewife	1.69 (1.31-2.17)*	1.44 (1.10-1.89)*
Other	1.09 (0.58-2.07)	0.73 (0.37-1.42)
**Wealth quintile**		
Lowest	1	1
Second	0.91 (0.69–1.19)	1.01 (0.76-1.33)
Middle	0.95 (0.71–1.26)	0.95 (0.70-1.28)
Fourth	1.44 (1.10–1.90)*	1.25 (0.93-1.67)
Highest	1.72 (1.30–2.27)*	1.38 (1.02-1.86)*
**Knowledge of danger signs**		
No	1	1
Yes	1.99 (1.67–2.38)*	1.71 (1.41-2.07)*
**Distance to health facility**		
> 30 min	1	1
≤ 30 min	1.69 (1.39-2.05)*	1.44 (1.18-1.77)*

*CI* confidence interval.

*p < 0.05.

Results are shown for the association between completed antenatal care visits (≥ 4 visits) and various independent variables in mid- and far-western Nepal (n = 2,190). Of the total study population (n = 2,481), only 2,190 women had completed at least one antenatal checkup during the preceding 12 months.

Similarly, women who knew at least one danger sign of pregnancy and delivery were 1.3 times more likely to use SBA services at delivery (OR = 1.31, 95% CI: 1.08-1.58). Four or more ANC visits was a determining factor for SBA utilization, and women who attended four or more ANC visits were 2.4 times more likely to use SBA services at delivery (OR = 2.39, CI: 1.97-2.89). Distance from a health facility contributed to delivery service utilization, and mothers who lived within 30 min or less from a health facility were 1.25 times more likely (OR = 1.25, CI: 1.03-1.52) to use SBA during delivery compared to those who lived further away. The odds that women would utilize SBA services during delivery were higher among those with a higher level of education and higher wealth quintiles compared to illiterate women and women in the lowest wealth quintile, respectively (Table 4).

**Table 4:** Determinants of delivery service utilization

**Characteristics**	**Skilled birth attendant utilization**	
	**Bivariate OR (95% CI)**	**MultivariateOR (95% CI)**
**Education**		
Illiterate	1	1
Informal education	1.49 (1.20–1.86)*	1.18 (0.92–1.51)*
Primary school	2.98 (2.25–4.00)*	1.72 (1.25–2.36)*
Secondary school	4.10 (3.17–5.31)*	2.05 (1.52–2.77)*
Intermediate and above	7.75 (5.30–11.33)*	4.41 (2.89–6.72)*
**Wealth quintile**		
Lowest	1	1
Second	0.81 (0.63–1.04)	1.08 (0.81-1.43)
Middle	0.99 (0.76–1.29)	1.19 (0.88-1.61)
Fourth	2.53 (1.96–3.26)*	1.99 (1.49-2.67)*
Highest	2.67 (2.07–3.46)*	1.90 (1.42-2.56)*
**Knowledge of danger signs**		
No	1	1
Yes	2.09 (1.78–2.45)*	1.31 (1.08-1.58)*
**Antenatal care visits**		
< 4 visits	1	1
≥ 4 visits	2.92 (2.44–3.51)*	2.39 (1.97-2.89)*
**Distance to health facility**		
> 30 min	1	1
≤ 30 min	1.87 (1.57–2.21)*	1.25 (1.03-1.52)*

*CI* confidence interval.

*p < 0.05.

Results are shown for the association between delivery service utilization and various independent variables in mid- and far-western Nepal (n = 2,481).

## Discussion

This study describes factors that influence women’s utilization of antenatal and delivery care services in rural Nepal and details the barriers to SBA utilization.

### Antenatal care services

Our results show that women who knew the danger signs of delivery and pregnancy had also completed four or more ANC visits, nearly twice as many as women who lacked such knowledge. Because we investigated these phenomena retrospectively, ANC visits may have increased the women’s knowledge and subsequently may have influenced ANC utilization.

The current study confirmed an association between distance and ANC utilization (i.e., mothers living 30 min or less from a health facility were 1.4 times more likely to use ANC services compared to those who had to travel more than 30 min). Similarly, an Ethiopian study reports that mothers who considered pregnancy a risky event were more likely to seek ANC services compared to those who considered pregnancy risk-free, and those who lived less than one hour from a health facility were four times more likely to use ANC than those who lived more than two hours away [22]. In our study area, people commonly walk to the health facility, although public transport (mostly buses and minibuses) serves health facilities near the major highway. Private transportation is rare. A study from Laos reports that women did not utilize ANC services due to normal pregnancy and/or feeling normal (49%), difficulty accessing the clinic (48%), and time restraints or being busy (14%) [23].

In our study, women with higher levels of education used ANC services more frequently than illiterate women or women with lower-level education. Women with secondary-level or higher education were approximately two times more likely to utilize ANC services than illiterate women. In rural India, adolescent women with middle- and higher-level education were two and three times more likely, respectively, to utilize full ANC services than uneducated women [24]. Indeed, women's education level is the best predictor of ANC visits [25].

### Delivery service utilization

Our results showed unequal service utilization regarding mothers’ education level, wealth quintile, knowledge of danger signs, ANC service utilization, and distance to a health facility. Women who completed four or more ANC visits utilized delivery services twice as often compared to women who completed fewer than four visits. This finding supports a previous study that demonstrated an association between antenatal check-ups and SBA utilization at delivery in two VDCs near Kathmandu [26]. The same study reported that women with secondary school education or above prefer giving birth in a health institution [26]. Recent demographic and health survey data from Nepal and Bangladesh also report a positive association between education and the use of delivery services [4,27]. Further, the bivariate and multivariate analyses in our study confirm similar associations. In multivariate analysis, women with intermediate-level education or higher were four times more likely to use SBA delivery services.

Distance to a health facility and inadequate transportation posed major barriers to SBA service utilization in our study. Nearly half (46%) of the women who did not use SBA services at delivery indicated that the distance to the health facility prevented them from using the services. Similarly, 21% of respondents did not utilize SBA due to inadequate transportation. Other studies also report that long distance to a health facility negatively affects the utilization of delivery services [28-31]. In Nepal’s rural Kavre district, 30% of respondents mentioned inadequate transportation as a major reason for not using SBAs [29]. However, limited use of SBAs is not always due to economic, geographic, cultural, and religious reasons but rather may depend on institutional issues. In Nepal, additional reasons for low utilization of SBA services include poor quality service, unavailability and inaccessibility of services, minimal staff support, lack of medicine and equipment, and deficient referral systems [32]. Sociocultural norms associated with seeking SBA services change over time. Long-term investments in training health workers may accelerate a change toward seeking SBA services. Nevertheless, cost and access remain important barriers to the use of healthcare facilities for giving birth [33].

In Bangladesh, mothers’ education, parity, regular exposure to the media, number of ANC visits, and distance to the nearest health facility influence a child’s birthplace [30]. A study in Afghanistan also shows an association between use of SBAs at delivery, wealth quintile, and distance to a health facility [31]. Our study yielded similar results regarding the association of SBA utilization with the number of ANC visits and distance to the health facility. Indeed, factors that associate most consistently with receiving skilled care include higher maternal age, low parity, maternal education, and higher household economic resources [34].

### Study limitations

In our study, only 2,481 women were available for interview in the sampled clusters, which nonetheless yielded a power of 79%. Because we asked respondents how many times they received ANC service during the previous 12 months, our study may reflect a limited recall bias that applies only to a single delivery event. We did not explore the roles of husbands and other family members, which may influence women’s use of SBA services. Additionally, our study was limited to the mid- and far -western regions of Nepal, which have low utilization of ANC and delivery services. Nevertheless, our findings are relevant for other regions in South-East Asia with low SBA utilization.

## Conclusions

Fewer than half of the women in our study delivered babies with the aid of SBAs, demonstrating a need for increased utilization of such services in rural and remote areas of Nepal. Distance and inadequate transportation to health facilities pose major barriers to SBA utilization. In addition to improved transport to healthcare facilities, advancing transport costs to pregnant women before they go to a healthcare facility for delivery will increase SBA utilization. Moreover, SBA utilization associates positively with women’s knowledge of pregnancy danger signs, wealth quintile, and completed ANC visits. Nepal needs to develop strategies that generate demand for SBA services and reduce financial, geographic and cultural barriers to those services.

## Abbreviations

ANC: Antenatal care; IMR: Infant mortality rate; MCH: Maternal and child health; MDG: Millennium development goals; MMR: Maternal mortality ratio; SBA: Skilled birth attendant; VDC: Village development committee; WHO: World Health Organization.

## Competing interests

The authors declare that they have no competing interests.

## Authors’ contributions

SO and GB participated in the conception and design of our research. BC conceptualized the paper, analyzed data, searched literature, and wrote the manuscript draft. AK made critical revisions to the paper. AP, MP, and NS provided statistical inputs. SM, BS, and MM provided comments in the structure and contents of paper. All authors read and approved the final manuscript.

## Pre-publication history

The pre-publication history for this paper can be accessed here:

http://www.biomedcentral.com/1472-698X/13/49/prepub
